# Public Awareness and Acceptability of PGT-M in Cancer Predisposition Syndromes

**DOI:** 10.3390/genes14112069

**Published:** 2023-11-12

**Authors:** Davide Calosci, Lisa Passaglia, Ilaria Gabbiato, Francesca Cartisano, Rebecca Affuso, Ugo Sorrentino, Daniela Zuccarello

**Affiliations:** 1 Clinical Genetics Unit, Department of Women’s and Children’s Health, University of Padova, Via Giustiniani 3, 35128 Padova, Italy; lisa.passaglia@studenti.unipd.it (L.P.); ilaria.gabbiato@studenti.unipd.it (I.G.); francesca.cartisano@studenti.unipd.it (F.C.); rebecca.affuso@studenti.unipd.it (R.A.); ugo.sorrentino@unipd.it (U.S.); 2 Department of Lab Medicine Unit of Clinical Genetics and Epidemiology, University Hospital of Padova, Via Giustiniani 3, 35128 Padova, Italy; daniela.zuccarello@unipd.it

**Keywords:** Cancer Predisposition Syndromes, prenatal diagnosis, fertility preservation, PGT-M

## Abstract

Cancer Predisposition Syndromes (CPSs), also known as Hereditary Cancer Syndromes (HCSs), represent a group of genetic disorders associated with an increased lifetime risk of developing cancer. In this article, we provide an overview of the reproductive options for patients diagnosed with CPS, focusing on the emerging role of Preimplantation Genetic Testing for Monogenic disorders (PGT-M). Specifically, we conducted a literature review about the awareness and acceptability of its application to CPSs. Based on the available data, the awareness of the applicability of PGT-M for CPSs appears to be limited among both patients and physicians, and a heterogeneous set of factors seems to influence the acceptability of the procedure. Our findings highlight the need for increasing education about the use of PGT-M for CPSs. In this context, guidelines developed by professional or institutional bodies would represent a useful reference tool to assist healthcare professionals in providing proper preconception counseling.

## 1. Background

Hereditary transmission or de novo occurrence of pathogenic variants in a specific subset of predisposing genes is associated with an increased lifetime risk of developing tumors. These hereditary disorders, which are collectively referred to as Cancer Predisposition Syndromes (CPSs), impose a high burden on patients in terms of reduced quality of life and life expectancy but also troubled reproductive prospects. Assisted reproduction technologies, such as Preimplantation Genetic Testing for Monogenic disorders (PGT-M), offer a viable alternative to willing patients by preventing the transmission of causative variants through generations. However, according to the literature, the knowledge of these technologies among patients, and even professionals, remains limited, and so does patients’ willingness to make use of them. This appears to be influenced by a complex interplay of clinical, reproductive, demographic, socio-cultural, ethical, and psychological factors. As members of the Italian Network of Preimplantation Genetic Testing (N.I.D.O.), our aim is to give an insight into this topic by providing an overview of CPSs, the available reproductive options for affected patients and a descriptive review of studies exploring the awareness and acceptability of PGT-M among patients and professionals and the potential influencing factors.

## 2. Cancer Predisposition Syndromes

Cancer Predisposition Syndromes (CPSs) are a heterogeneous group of genetic disorders characterized by an increased risk of developing cancer compared to the general population.

While most tumors arise sporadically as a consequence of acquired variants in somatic cells, 5–10% of cases are estimated to develop in patients affected by CPS. Typically, these tumors display one or more of the following characteristics: early age of onset; more than 1 tumor in the same individual; positive family history [1].

Knudson’s theory (the “two-hit hypothesis”) is the most widely accepted model for the pathogenesis of tumors in these patients [2]. According to this hypothesis, in somatic cells, pathogenetic variants are required to occur on both alleles of the same oncosuppressor gene to allow a tumor to develop. Therefore, the constitutional presence of an inherited genetic variant (a so-called “germline” variant) in a cancer predisposition gene predictably increases these patients’ lifetime risk of developing tumors.

Certain features can serve as red flags for suspecting a diagnosis for different syndromes, such as:

- onset of tumors that are extremely rare in sporadic form (e.g., endolymphatic sac tumors, associated with VHL Syndrome [3]; pleuropulmonary blastoma, associated with variants in *DICER1* [4]);
- onset of tumors with specific histological characteristics (e.g., triple-negative breast cancer tumors and serous ovarian adenocarcinomas, both frequently associated with variants in *BRCA1/2* [5]);
- origin from specific geographical areas (e.g., the high prevalence of a specific variant in *SDHD* in Trentino, Italy [6]; updated clinical practice guidelines for Diffuse Gastric Cancer (DGC) recommend that all New Zealand Māori with a confirmed diagnosis of DGC should undergo genetic testing, given the high prevalence of *CDH1* variants in this population [7]).
- coexistence of other clinical manifestations, not necessarily neoplastic (e.g., dysmorphic features in patients with Cowden Syndrome [8]; café-au-lait spots in patients with Neurofibromatosis 1 [9]).

More than 50 CPSs have been described in the literature so far. A description of the most frequent ones is provided in Table A1.

The diagnosis of a CPS has significant clinical consequences for these patients. First of all, it allows access to targeted surveillance programs, which aim to diagnose a tumor at its early stages, start timely specific therapies and improve patients’ prognoses.

For some syndromes, primary prevention interventions are also possible (e.g., prophylactic colectomy in patients with FAP, or breast and/or ovarian removal in patients with *BRCA* variants) [10,11]. Secondly, tumors with alterations in certain genes are more responsive to specific chemotherapy drugs (e.g., *BRCA*-associated tumors and PARP inhibitors) [12]. In addition to individual benefits, the diagnosis also has repercussions on patients’ family members. In most cases, CPSs are inherited in an autosomal dominant manner, which means that each affected individual has a 50% chance of transmitting the causative variant to their offspring [13]. If the causative variant is known, it can be searched for in other family members, ensuring that they receive the same aforementioned preventive/therapeutic measures. Knowing the genetic cause can also be important for patients planning a pregnancy.

## 3. Reproductive Choices Available to Patients Affected by CPSs

There are different reproductive options, approved by the Ethics Committee of the American Society for Reproductive Medicine, available for CPS patients who are planning a pregnancy [14].

In case of spontaneous pregnancy, couples can opt for invasive prenatal diagnosis (via chorionic villus sampling or amniocentesis) to research the inherited causative variant in the fetus and potentially consider a voluntary termination of pregnancy (VTP).

In general, invasive prenatal diagnosis is considered more acceptable for early childhood cancers with high penetrance and no prevention/treatment available (e.g., Li Fraumeni syndrome) than it is for low-penetrance or later-onset cancers with an effective method of prevention/treatment [15].

Otherwise, in recent years Preimplantation Genetic Testing (PGT) on embryos at the blastocyst stage, as part of a medically assisted fertilization process, has been steadily rising in priority [16]. However, the utilization of PGT for CPSs is still constrained by various factors. These include insufficient awareness among medical professionals regarding pre-implantation genetic tests, limited understanding of their applicability [17], an ongoing ethical debate surrounding the appropriateness and acceptability of the procedure for hereditary oncological conditions [16] and challenges related to the accessibility and costs associated with PGT-M programs; these challenges encompass economic considerations as well as the psychological impact on individuals [18,19].

Another possibility, specifically for women with a reduced ovarian reserve or who have not made use of fertility preservation procedures, is egg donation [16].

### 3.1. Preservation of Fertility

Patients diagnosed with CPS have an increased risk of becoming infertile, because of the disease itself or as a consequence of medical or surgical therapies that could damage germ cells. Informing these patients about procedures aimed at preserving fertility is recommended at the time of diagnosis.

For female patients, the most common fertility preservation techniques are ovarian tissue and oocyte cryopreservation. Ovarian tissue cryopreservation in prepuberal girls or adolescents with CPS-associated cancer has been proposed as an effective method by Mertes et al. in 2015 [20]. In the past, this option was also offered to women with cancer who needed to start chemotherapy as soon as possible, as they did not have the time for ovarian stimulation necessary for oocyte cryopreservation. Thanks to the new hormonal stimulation techniques, which allow the procedure to start on any day of the menstrual cycle (RANDOM-START protocol), oocyte cryopreservation is preferred in all women after puberty. This technique involves controlled ovarian stimulation, which lasts for about 14 days and requires having reached puberty [21,22]. During controlled ovarian stimulation, circulating estrogen levels can increase up to 10 times their physiological levels. In patients with hormone-sensitive tumors, the use of aromatase inhibitors during ovarian stimulation has been shown to increase the response to ovarian stimulation and reduce circulating estradiol levels. There has been no evidence of a significantly increased risk of cancer recurrence in these patients, both in the short and long term [21,22].

Male fertility preservation has also been increasingly made available in the last two decades. Adequate counseling before initiating therapies with potential gonadotoxic effects is essential; in fact, chemotherapy and radiotherapy can cause testicular failure and ejaculatory dysfunction. The tumors with the greatest impact on seminal fluid parameters are prostate cancer and testicular cancer [23].

The gold standard for male fertility preservation is sperm cryopreservation, which has become an essential part of Assisted Reproductive Techniques (ARTs). Patients must be adequately informed about the potentially higher risk of “genetic” damage if semen is collected after treatment has started, therefore semen should be collected prior to initiation of treatments [24]. Other methods to preserve male fertility, such as cryopreservation of testicular tissue and testicular tissue re-implantation or grafting, are currently performed only in approved clinical trials or experimental protocols [24].

### 3.2. Invasive Prenatal Diagnosis

Invasive prenatal diagnosis includes a set of procedures aimed at taking a sample of embryo-fetal or adnexal tissues, in order to investigate suspected chromosomal anomalies or monogenic pathologies [25], with CPSs falling into the latter category.

In the case of spontaneous pregnancy, the identification of the causative variant allows the use of invasive prenatal diagnosis, in order to search for this variant in DNA extracted from placental or fetal material.

Currently employed invasive prenatal diagnostic techniques are based on chorionic villus sampling or amniotic fluid sampling. The choice of the technique depends on the indication, the time of sampling, the specific experience of the operator and reference laboratory, as well as the preferences of the adequately informed woman.

Chorionic villus sampling is performed between the 10th and 12th week of pregnancy. It involves ultrasound-guided transabdominal sampling of trophoblast cells [26]. The risk of spontaneous abortion, linked to the invasiveness of this technique, is estimated to be 1:500 [27] but varies significantly depending on the experience of the operator. The tissue acquired by sampling can be used for cytogenetic analysis on cytotrophoblast cells or cultures (chorionic villus mesenchymal cells), as well as for molecular investigations (e.g., search for familial variants in genes associated with predisposing tumor syndromes) [25]. The advantage of the precocity of the technique, compared to amniocentesis, is counterbalanced by its greater invasiveness and the sampling of placental, rather than fetal, tissue.

Amniocentesis is an invasive prenatal diagnostic technique based on the collection of amniotic fluid around the 15–18th gestational week [25]. The risk of spontaneous abortion, related to the invasiveness of the technique, is approximately 1:1000 [27]; however, as in the case of chorionic villus sampling, the experience of the operator is a crucial variable. The amniotic fluid is composed of a non-corpuscular component, devoid of cells, and a corpuscular component, represented by amniocytes, which derive from the flaking of cells of the skin, mucous membranes, genitourinary tract, gastrointestinal tract of the fetus, and from amniotic membranes. Amniocytes can be used for cytogenetic and molecular investigations, as well as for biochemical analyses [25]. Furthermore, on the non-corpuscular portion, it is possible to dose alpha-fetoprotein (AFP) and other biochemical markers.

### 3.3. PGT-M

The identification of the molecular cause of a specific syndrome allows Preimplantation Genetic Testing (PGT). PGT is a technique that enables the identification of genetic alterations in embryos obtained through in vitro fertilization techniques. In 2017, the term PGT replaced the previous nomenclatures of Preimplantation Genetic Diagnosis (PGD) and preimplantation genetic screening (PGS) [28]; in this article, we will use the term PGT.

The first case of a child born following PGT was reported in the early 90′s by Handyside et al., who described the use of PCR to select female embryos in families carrying recessive X-linked disorders (mental retardation and adrenoleukodystrophy) [29].

Over the years, PGT has evolved from an experimental procedure to a commonly used technique in clinical practice, as an alternative to invasive prenatal diagnosis for couples planning a pregnancy [30]. Different protocols have been proposed and validated, each with its own merits and limitations, but the fundamental steps of the technique generally involve the biopsy of one or more cells from the embryo, the search for any genetic abnormalities in the sample and the selective transfer of alteration-free embryos into the mother’s uterus [31,32].

PGT-M is a specific type of PGT that allows the detection of causative variants of monogenic disorders. Its ultimate goal is to enable patients to have children unaffected by the same condition and to avoid the risk of adverse outcomes associated with invasive prenatal diagnosis (miscarriage) and the burden associated with pregnancy termination in the event of a pathological result.

The first documented case of the application of PGT-M for CPSs was reported by Verlinsky et al. in 2001. They described the case of a healthy child born after PGT-M from a couple with a paternal family history of Li–Fraumeni syndrome [33]. Verlinsky et al. also described the use of the same technique in couples diagnosed with Neurofibromatosis 1 and Neurofibromatosis 2 (currently known as *NF2*-related Schwannomatosis) [34]. In 2002, Rechitsky et al. extended the technique to couples with Familial Adenomatous Polyposis (FAP), Von-Hippel–Lindau (VHL) Syndrome, Retinoblastoma, and predisposition to *SMARCB1*-associated brain tumors [35].

Data collected by ESHRE for the decade 1997–2007 showed a progressive increase in the number of PGT cycles for CPSs. The most common indications were high-penetrance conditions (primarily Neurofibromatosis 1, Familial Adenomatous Polyposis and von Hippel–Lindau Syndrome); however, lower-penetrance forms, such as HBOC Syndrome, were also showing an emerging pattern [36]. Conversely, the latest ESHRE data collection, published in April 2023 and referring to 2018, reports that preimplantation analysis for *BRCA1* has become the second most frequent indication to perform PGT-M (5.4% of the total, after Huntington’s disease 9%), followed by Neurofibromatosis 1 in seventh place [37].

A recent review by Vriesen et al. reports that success rates (in terms of CPR, Clinical Pregnancy Rate, and LBR, Live Birth Rate) for CPSs are similar to those of other monogenic diseases [38].

However, ethical issues have been raised about the legitimacy of applying PGT-M for CPSs [30]. The reasons are mainly related to the incomplete penetrance and variable expressivity of these conditions, as well as the availability of surveillance programs aimed at reducing the mortality and morbidity of affected patients. It is noteworthy that in 2003 the ethical task force of ESHRE labeled the use of PGT-M for studying causative variants of late-onset diseases and multifactorial diseases, including CPSs, as “acceptable”. In the United Kingdom, the Human Fertilisation and Embryology Authority (HFEA) has approved the use of PGT for the diagnosis of some CPSs, including HBOC, Li–Fraumeni, and FAP (the complete list can be found online: https://www.hfea.gov.uk/pgt-m-conditions/?page=67, accessed on 31 July 2023). Moreover, since PGT does not expose the couple to the risk of VTP (Voluntary Termination of Pregnancy) it may be ethically and psychologically better accepted than prenatal diagnosis techniques.

However, some studies highlight there is still poor awareness among doctors of the applicability of PGT-M for CPSs. It would be important to evaluate whether its limited use is due to patients’ choices or the lack of knowledge among doctors about this diagnostic option [39].

In this descriptive review, we studied the available literature on the application of PGT-M for CPSs, focusing on its perception by both physicians and affected individuals and on the reasons why its use for these syndromes is still limited to date. Pertinent English language articles were searched in PubMed (https://pubmed.ncbi.nlm.nih.gov/, last accessed on 31 July 2023). Searched terms included PGT-M and its prior nomenclature (i.e., preimplantation genetic diagnosis [PGD]), combined with terms related to hereditary tumors (e.g., hereditary neoplastic syndrome, HBOC, Lynch Syndrome, …). 

## 4. Knowledge of PGT

Articles about the use of PGT for CPSs consistently report the stance that patients should be systematically informed about this diagnostic technique, regardless of the specific syndrome [40,41]. This opinion seems to be shared by both healthcare providers and patients.

However, various studies have also highlighted that patients’ awareness of this reproductive option is still limited. In 2012, Quinn et al. published a meta-analysis of studies conducted between 1992 and 2009 on the use of PGT for CPSs. 7 articles concerned the application of PGT for Hereditary Breast and Ovarian Cancer syndrome, 2 for Familial Adenomatous Polyposis, 1 for Von Hippel–Lindau syndrome, 1 for Li–Fraumeni syndrome, and 3 for genetic predisposition to cancer in general. In this meta-analysis, only 35% of patients were aware of the applicability of PGT to CPSs [41]. In Rich et al.’s case series involving patients with MEN1, MEN2, FAP, Lynch syndrome and HBOC syndrome the percentage was 24% of 370 adults diagnosed with a CPS [1].

More recently, in Villy et al.’s retrospective study, 28 patients with different CPSs were asked how they became aware of the possibility of undergoing PGT. Of them, 75% had received the information from a physician (in two-thirds of cases, from a geneticist). The cohort was mainly composed of patients with VHL syndrome (9/28), Familial Adenomatous Polyposis (8/28), and *CDH1*-related gastric tumor predisposition (5/28), followed by patients with variants in *STK11* (2/28), *AXIN2*, *BRCA1*, *MEN1*, and *FH* (1 patient for each gene) [40].

Socioeconomic status emerged as a factor influencing patients’ awareness of the technique [40]. An income-related difference was also reported by Rich et al. (12% of patients with income below 20 K $ were aware of the technique, compared to 38% of those with 20–50 K and 22% for >100 K $) [1]. Based on these data, it could be hypothesized that information is more accessible to higher-income classes; however, according to the authors, the motivation can also be attributed to different approaches by physicians [1,42].

Regarding healthcare providers, in 2010 Brandt et al. addressed a survey of professionals involved in the management of patients affected by CPSs, such as gynecologic oncologists, obstetricians, and gynecologists, assessing their knowledge of the topic and their personal perception and experience with the application of PGT for HBOC and FAP syndromes. The questionnaire revealed that 68% of them had limited or incorrect knowledge about the use of PGT for CPSs, while over 80% expressed their willingness to refer these patients to professionals experienced in PGT [43].

**Scheme 1:**
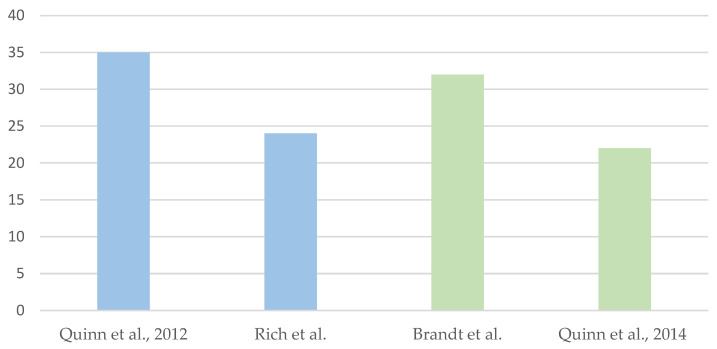
Percentage of patients (blue) and healthcare providers (green) who were aware of the applicability of PGT-M to CPSs [1,41,43,44].

The desire for further education on PGT is not limited to physicians, as shown in the 2014 study by Quinn et al. targeting nurses from the Moffitt Cancer Center. The study found that 78% declared not to be familiar with PGT, but more than half were in favor of its use for CPSs [44]. Scheme 1 provides a graphical representation of the aforementioned data.

## 5. Acceptability of PGT

### 5.1. Healthcare Providers’ Opinion

The limited available studies on CPSs show that the acceptability of PGT to professionals is often influenced by the type of syndrome and the oncological history of patients.

For example, a 2009 French study investigated the levels of acceptability of PGT among geneticists, revealing a correlation with the clinical characteristics of the different diseases. Specifically, for syndromes with childhood-onset, multifocal presentation, high penetrance and limited prevention and/or treatment options, the technique was considered acceptable by 76.3% of respondents. However, the percentage dropped to 13.2% for syndromes with adult-onset, localized presentation, high penetrance, effective prevention and/or treatment options, but with impact on quality of life. Furthermore, it reached 0% for syndromes with similar characteristics but with preserved quality of life. In order, the syndromes for which PGT was considered acceptable were: Li–Fraumeni syndrome (67.1% of respondents), retinoblastoma (47.4%), familial adenomatous polyposis (39.5%), MEN2A (11.8%), neurofibromatosis type 1 (11.8%), HBOC (7.9%), HNPCC (6.6%) [15].

More recently, a Dutch study showed high levels of approval regarding the application of PGT for individuals carrying *BRCA* variants (>85% of respondents, including geneticists, gynecologists, and oncologists). In particular, 92% stated that they would propose this solution to their future patients. The authors attribute this trend to the increasing development of PGT in recent years. However, most professionals still reported low to moderate knowledge of the technique [45].

### 5.2. Patients’ Opinion

**Scheme 2:**
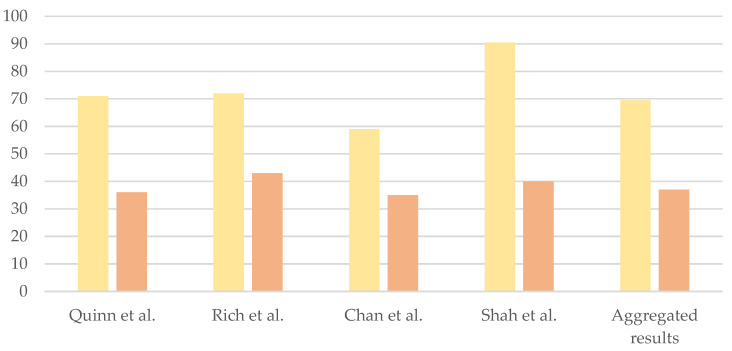
Comparison between the percentage of patients who think that PGT should be offered as a reproductive option for couples affected by CPSs (yellow) vs. percentage of patients who would consider PGT for themselves (orange) [1,41,46,47].

The meta-analysis by Quinn et al. revealed that the majority of interviewed patients (71%) believed that PGT should be offered as a reproductive option for couples affected by CPSs, but only 36% would use it personally [41]. This discrepancy is a recurrent finding in the scientific literature (72% vs. 43% in Rich et al.’s study; 59% vs. 35% in Chan et al.’s case series involving female patients with *BRCA1*/*2* variants) [1,46] (Scheme 2). In Shah et al.’s study targeting patients with *CDH1* variants, 40% of respondents stated they would consider PGT, 35% would not and 25% were uncertain; 90.5% of patients thought that physicians should discuss PGT with individuals carrying *CDH1* variants [47].

A heterogeneous set of factors that might influence the acceptability of PGT among patients is reported to date. We classified them into four groups: clinical factors (related to the specific condition), demographic factors, reproductive factors, and one last group including sociocultural, ethical and psychological factors (a summary of these factors is provided in Table 1).

#### 5.2.1. Clinical Factors

The study by Rich et al. aimed at comparing the opinions of cohorts of patients affected by different syndromes (MEN1, MEN2, FAP, Lynch syndrome, and HBOC). The purpose was to highlight potential syndrome-specific factors that might influence the acceptability of PGT. The percentage of patients considering PGT was higher among those affected by MEN1 and FAP, while it was lower for patients diagnosed with MEN2 [1].

According to Hansen et al., the low interest in PGT among patients with MEN2 (especially MEN2A) might be attributed to the existence of prophylactic thyroidectomy as a primary preventive intervention for medullary thyroid carcinoma. However, it is essential to point out that this intervention carries surgical risks and does not protect against other clinical manifestations of the condition (e.g., the development of pheochromocytoma) [48].

Higher levels of acceptability were observed in subjects affected by early-onset diseases and in subjects with conditions for which prophylactic surgery is not available. Higher levels of acceptability were also reported for syndromes with a perceived higher disease burden [1].

#### 5.2.2. Demographic Factors

Regarding demographic factors, Rich et al. found that the only factor influencing the acceptability of PGT was gender, with the highest levels among male subjects. One reason hypothesized by the authors was the concern of female patients regarding the exposure of cancerous or precancerous cells to high estrogen levels. In fact, while IVF techniques for males involve gamete donation, females undergo ovarian stimulation treatments to induce ovulation, as well as procedures for egg retrieval and subsequent embryo transfer [1]. Differently, age, ethnicity, income, and education were not found to be significant factors conditioning respondents’ opinions.

The survey conducted by Krones et al. in Germany yielded the following findings: favorable attitude towards PGT for CPSs was reported by 59.4% of male respondents and 55.8% of female respondents; regarding the percentage of respondents open to considering PGT, 40.1% of males and 32.2% of females responded positively [49].

The limitation of this survey is that it polled the general population and not patients (as emphasized by Rich et al., the personal experiences of these patients play a key role in modifying their perception of the procedure). The article by Krones et al. also compared the percentage of individuals in favor of PGT for CPSs in different countries, showing 50% of respondents in favor in Germany and 60% in the USA [49]. In the study by Marteau et al. on the British population, the percentage dropped to 34% (although it should be noted that this study was published 10 years earlier than Krones et al.’s) [50].

Regarding HBOC syndrome specifically, a review by Lombardi et al. in 2022 confirmed higher levels of acceptability among male subjects [42]. Moreover, carrier status for HBOC syndrome correlated with a reduced desire for parenthood among female subjects, but not among male subjects. This same difference was also observed with regard to the desire to have a new pregnancy in *BRCA1/2* patients with previous children.

#### 5.2.3. Reproductive Factors

In the study by Rich et al. CPS couples with children, as well as couples accepting VTP as an option, appeared to have a lower degree of acceptability of PGT. In this regard, the authors suggest that parents may have concerns about the psychological consequences on a firstborn affected by a CPS if they decide to have another child who will not be affected because of PGT [1].

In the case series of *BRCA* carriers by Chan et al., a comparison of attitudes towards PGT in women whose families were regarded as complete vs. not complete showed similar results [46].

In Shah et al.’s study, of the two *CHD1* carriers who wanted to have a biological child, one was “very likely” to use PGT, whereas the other one was “very unlikely”. Among respondents who already had children, a small majority indicated that they would have considered using PGT if it had been available [47].

#### 5.2.4. Socio-Cultural, Ethical and Psychological Factors

In the 2021 review by Hughes et al. regarding elements influencing patients’ decisions about Preimplantation Genetic Testing, a complex set of factors emerged, including ethical and religious ones [18].

Some patients reported that they feel a sense of responsibility when considering PGT as an option, while others showed reluctance due to their religious beliefs. Similar considerations emerged from a cross-sectional study by Shah et al. targeting patients with a genetic predisposition to develop gastric tumors. Out of 38 investigated patients, 13 identified their “philosophy of life” as an influencing factor, followed by “God, religion, and morality” (10/38), the “desire to eradicate the variant for future generations” (9/38), and the desire to “minimize anxiety or suffering,” both on a personal level and for their offspring (5/38) [47].

In the investigation by Rich et al., religious patients were less favorable to the question of whether PGT should be offered compared to those who defined themselves as non-religious (69% versus 89%) [1]. According to the review by Lombardi et al., two studies stated that religious beliefs did not influence the choice of resorting to assisted reproductive techniques [42]. Conversely, Menon et al. found that women who had opted for PGT tended to be less religious [51].

In many cases, PGT may be considered an acceptable option when pregnancy termination or gamete donation is not compatible with certain ethical and religious beliefs [48]. In Kastrinos et al. case series, avoiding a potential pregnancy termination was regarded as important by 64% of women and 71% of men [52]. Lammens et al. reported that avoiding a potential termination of pregnancy was the most frequently perceived advantage of PGT among patients (32% of the total) [53].

Similarly, in the study by Derks-Smeets et al., none of the 6 couples who underwent PGT considered invasive prenatal diagnosis acceptable; specifically, all couples perceived a “moral difference” between embryo selection and pregnancy termination, considering termination too drastic of a solution [54].

In the meta-analysis by Quinn et al., the most frequently reported factor influencing the acceptability of PGT was couples’ concern for the health of the newborn. However, most studies also showed that 33% of respondents reported ethical concerns regarding the use of PGT, including those who expressed a favorable view of the procedure [41]. Derks-Smeets et al. also reported that protecting the child from the variant was one of the main psychological factors that led couples to choose PGT; factors against PGT included the fear of a loss of romance in the relationship, having to resort to IVF techniques, and the percentage of procedure failures [54].

Another key element to consider is the personal experience of these families. This includes not only the oncological history of the patient but also the one of affected family members. For example, in Kastrinos et al.’s study on patients with FAP, the main factors reported to influence a tendency toward prenatal diagnosis (invasive/preimplantation) were: already having a child affected with cancer and experiencing the loss of a family member secondary to FAP-associated cancer. In this case series, 19 out of 20 FAP patients would consider prenatal diagnosis (specifically, 90% would consider PGT and 75% would consider invasive prenatal techniques), and all of them deemed it ethical to offer PGT to affected families [52].

Regarding personal oncological history, Fortuny et al. [55] and Menon et al. [51] reported a greater tendency to consider PGT among patients with a previous diagnosis of tumor. Derks-Smeets et al. [54], Gietel-Habets et al. [45], and Ormondoryd et al. [56] found that the most important factor influencing couples’ reproductive decisions was their personal and family history of cancer.

## 6. Discussion

From our review of the literature, it emerged that the number of studies concerning the application of PGT for CPSs is still limited.

It should be noted that the reported data span over a period of more than 20 years and refer to studies conducted in various countries with different legislation regarding the applicability of PGT. These factors may limit the interpretation of the available information, for example when comparing data of patients from different cohorts.

Regardless of the specific syndrome, the awareness of the applicability of PGT for CPSs appears to be still limited, among both health professionals and patients.

Several factors seem to influence physicians’ willingness to inform patients about this technique, primarily the characteristics of the specific pathology. According to Rich et al., physicians may also be reluctant to propose PGT to families they perceive as unable to afford it [1]. However, it is a recurring opinion in the literature that the decision to consider this reproductive choice should be up to patients. Additionally, the fact that patients already have children or do not explicitly express a desire for parenthood should not prevent the professional from providing them with all relevant information. As these conditions are hereditary, patients may share useful information with other affected relatives and advise them to undergo genetic counseling. It should be emphasized that these considerations apply not only to geneticists; all clinicians involved in the management of patients diagnosed with CPS should be able to identify patients eligible for PGT and refer them to the appropriate specialists.

Studies focusing on patients’ opinions have highlighted a different set of factors influencing the acceptability of the procedure, including psychological factors closely related to their personal and familiar experiences. Most patients believe that PGT should be systematically offered as a reproductive option, but the proportion of those who would use it themselves is generally lower.

Once again, it is crucial for professionals to be aware of the complexity of the decision-making process for couples considering Assisted Reproductive Technologies (ARTs). During counseling, psychological factors should also be taken into account. Therefore, multidisciplinary counseling, involving the geneticist, oncologist and psychologist should be considered for these syndromes. The ultimate goal is to support patients in making informed decisions by providing them with all the necessary information about the different reproductive options available.

Another remarkable point is that the expanding use of genetic testing for CPSs (for example, as a cascade testing in affected families) will lead to the identification of a greater number of patients of reproductive age who test positive for a CPS variant. As a result, we expect that in the near future, there will be an increasing number of patients who will have to face this complex decision-making process.

Based on these considerations, guidelines developed by professional or institutional bodies would represent a useful reference tool for healthcare professionals. Such guidelines should include reproductive counseling as an integral part of the care management of patients with a CPS (in this regard we point out that the latest guidelines for the management of medullary thyroid carcinoma recommend that patients diagnosed with MEN2A should be informed about the possibility of PGT) [57].

## Figures and Tables

**Table 1:** Summary of factors that may influence acceptability of the use of Preimplantation Genetic Testing for Cancer Predisposition Syndromes.

Clinical Factors	Demographic Factors	Reproductive Factors	Other Factors
Early age of onset	Male gender	Having previous children	Concerns for the newborn child’s health
High penetrance			Personal and familiar oncological history
High burden			
Availability of primary prevention measures		Willingness to consider PND + VTP	Ethical/moral beliefs
Availability of secondary prevention measures			Religious beliefs
Availability of effective treatment options			Concerns related to the procedure

The upper section of the table (green) shows those factors that seem to be associated with higher acceptance rates. The lower section (red) shows those factors that seem to be associated with lower acceptance rates.

## Data Availability

No new data were created or analyzed in this study. Data sharing is not applicable to this article.

## Appendix A

**Table A1:** Cancer Predisposition Syndromes.

Syndrome	Genes	H ^1^	Prevalence	Tumor Site	Lifetime Risk	Mean Age Onset
Lynch	*MLH1* *MSH2* *MSH6* *PMS2* *EPCAM*	AD	1:279	Colorectal, endometrial, ovarian, gastric and duodenal, distal small bowel, urinary tract (renal pelvis, ureter, and/or bladder), pancreatic	22–78% F 22–71% M	43–69
Constitutional Mismatch repair deficiency	*MLH1* *MSH2* *MSH6* *PMS2*	AR	-	Brain, digestive tract, hematological, Lynch syndrome associated, others	33–50%	1–65
FAP	*APC*	AD	From 1:6850 to 1:31,250 live births	Colon-rectal, small bowel (duodenum, most often periampullary region, or distal to the duodenum), pancreatic, thyroid, CNS, liver, bile ducts, gastric	70%	34–43
Peutz-Jeghers	*STK11*	AD	From 1:25,000 to 1:280,000	Colorectal, gastric, small bowel, breast, ovarian (mostly SCTAT), cervix (adenoma malignum), uterine, pancreatic, testicular (Sertoli cell tumor), lung	7–54%	6–59
MUTYH associated polyposis	*MUTYH*	AR	1–2% het. 1:20,000 to 1:60,000 biallelic	Colorectal, duodenal, ovarian, bladder, breast, endometrial, gastric, pancreatic, skin, thyroid	80–90% colorectal 1–25% others	38–61
Hereditary diffuse gastric cancer	*CDH1*	AD	1–3%	Lobular breast cancer, colorectal (uncertain)	56–70% hdgc 42% lbc in female	14–69 (average 38)
HBOC	*BRCA1* *BRCA2*	AD	1:400–1:500	Breast, contralateral breast, ovarian, male breast, prostate, pancreatic, melanoma (cutaneous and ocular)	1–3% for male breast and pancreas 21–72% others	44–48
VHL	*VHL*	AD	Between 1 in 31,000 to 1 in 91,000	CNS hemangioblastoma, retinal hemangioblastoma, renal cell carcinoma, pheochromocytomas, pancreatic cyst and neuroendocrine tumors, endolymphatic sac tumors, epididymal or broad ligament papillary cyst adenomas	10–70%	1–78
Li-Fraumeni	*TP53*	AD	1:3555 to 1:5476 data not well established	Adrenocortical, breast, CNS, osteosarcomas and soft-tissue sarcomas. Several additional cancers including leukemia, lymphoma, gastrointestinal cancers, cancers of head and neck, kidney, larynx, lung, skin (e.g., melanoma), ovary, pancreas, prostate, testis, and thyroid	≥70% for men and ≥90% for women	Any age
NF1	*NF1*	AD	1:2052 between ages 0 and 74 years	Optic pathway glioma, non-optic glioma, malignant peripheral nerve sheath tumor, Breast cancer, rhabdomyosarcomas, pheochromocytomas, paragangliomas, gastrointestinal stromal tumors, glomus tumors	2–20%	Birth-any age
Schwannomatosis	*SMARCB1* *LZTR1* *NF2*	AD	1/70,000	Schwannomas, meningiomas, MPNST	-	Any age
DICER1 Tumor Predisposition	*DICER1*	AD	1/5000	Pleuropulmonary blastoma (PPB), pulmonary cysts, thyroid gland neoplasia, ovarian tumors	Lung cysts/type Ir PPB in 25–40%; PPB types I, II, & III in <10%	Any age
Cowden	*PTEN*	AD	Unknown but is estimated at 1/200,000	Thyroid, breast, kidney, and endometrium	Breast cancer is 85%, thyroid cancer approximately 35% renal cell cancer 34% endometrial cancer 28%	38 and 46
PPGLs	*MAX* *SDHA* *SDHAF2* *SDHB* *SDHC* *SDHD TMEM127*	AD	1-9/1,000,000	Paragangliomas, pheochromocytomas, GISTs, pulmonary chondromas, renal clear cell carcinoma, papillary thyroid carcinoma, pituitary adenomas, and neuroendocrine tumors	-	Childhood
MEN1	*MEN1*	AD	Between 1:10,000 and 1:100,000	Parathyroid tumors, pituitary tumors, well-differentiated endocrine tumors of the gastro-entero-pancreatic tract, carcinoid tumors, adrenocortical tumors	-	20–25
MEN2	*RET*	AD	1:35,000	Medullary Thyroid carcinoma, pheochromocytoma, Parathyroid Disease	20–100%	30–70
Retinoblastoma	*RB1*	AD	1:15,000 and 1:20,000	Retinoblastoma, retinoma, pinealoblastomas, osteosarcomas, soft tissue sarcomas (mostly leiomyosarcomas and rhabdomyosarcomas), or melanomas	-	1–5
Gorlin	*PTCH1* *SUFU*	AD	Nearer to 1:30,827 A study in Australia gave a minimum prevalence of 1:164,000	Medulloblastoma, basal cell carcinoma, cardiac and ovarian fibromas, rhabdomyomas	2–20%	Adolescence-30
Carney complex	*PRKAR1A*	AD	Unknown	Myxomas, primary pigmented nodular adrenocortical disease (PPNAD), growth hormone (GH)-producing adenoma, large-cell calcifying Sertoli cell tumors (LCCSCT), thyroid adenoma or carcinoma, psammomatous melanotic schwannoma (PMS), breast ductal adenoma	-	Birth-4 decade
Familial melanoma syndrome	*CDKN2* *ACDK4* *BAP1* *POT1* *TERF2IP* *ACD* *TERT* *MITF* *MC1R*	AD/M ^2^	Unknown	Pancreatic, melanoma, others	-	30–40
Birt-Hogg-Dubè	*FLCN*	AD	More than 400 affected families from various populations have been described	Cutaneous manifestations (fibrofolliculomas, acrochordons, angiofibromas, oral papules, cutaneous collagenomas, and epidermal cysts), pulmonary cysts/history of pneumothorax, and various types of renal tumors	-	Childhood-69
Ataxia teleangectasia	*ATM*	AR	In the US is 1:40,000–1:100,000	Leukemia, Lymphoma, ovarian cancer, breast cancer, gastric cancer, melanoma, leiomyomas, and sarcomas	38%	Variabile
Hereditary leiomiomatosis	*FH*	AD	-	Uterine leiomyomata Uterine leiomyosarcoma Cutaneous leiomyomata Cutaneous leiomyosarcoma Renal cell carcinoma	15–90%	-

^1^ Heritability ^2^ Multifactorial. Data sources: see Refs. [58,59,60,61,62,63].
